# Combination of polyhexamethylene guanidine hydrochloride and potassium peroxymonosulfate to disinfect ready-to-eat lettuce

**DOI:** 10.1039/d0ra08356a

**Published:** 2020-11-05

**Authors:** Jiayi Wang, Yougui Yu, Yuemei Dong

**Affiliations:** College of Food and Chemical Engineering, Shaoyang University Shaoyang 422000 China jiayiwangsyau@syau.edu.cn; Shijiashike Co., Ltd. Liaoyang 111000 China

## Abstract

There is increasing demand for improved fresh produce disinfection technology during the ready-to-eat stage, especially in low-income developing countries. We previously reported that polyhexamethylene guanidine hydrochloride (PHMG) is an effective sanitizer using fresh-cut lettuce as a model. As a low-cost alternative, in the present study, we examined the disinfection efficacy of combining PHMG with the oxidizing sanitizer potassium peroxymonosulfate (PMS). PHMG (150 mg L^−1^) reduced the counts of *Escherichia coli* O157:H7, non-O157 *E. coli*, *Listeria monocytogenes*, *Salmonella typhimurium*, and naturally present microbes on ready-to-eat lettuce. The disinfection efficacy of PMS was significantly lower than that of PHMG; however, the efficacy of their combination (100 mg L^−1^ PHMG + 50 mg L^−1^ PMS, 50 mg L^−1^ PHMG + 100 mg L^−1^ PMS, and 50 mg L^−1^ PHMG + 50 mg L^−1^ PMS) was equivalent to that of PHMG alone. Color and sensory analyses (crispness, color, flavour, and off-odor) indicated that the combination of PHMG and PMS will not lead to additional quality loss when compared with tap water treatment, and electrolyte leakage analysis showed no additional lettuce surface damage of the combination when compared with PHMG-only treatment. These results show that partial replacement of PHMG by PMS is a cost-reducing strategy, providing a theoretical foundation for its practical application.

## Abstract 2

Combination of polyhexamethylene guanidine hydrochloride and potassium peroxymonosulfate can achieve consistent disinfection effects as those obtained with polyhexamethylene guanidine hydrochloride but at a lower cost.

## Graphical abstract

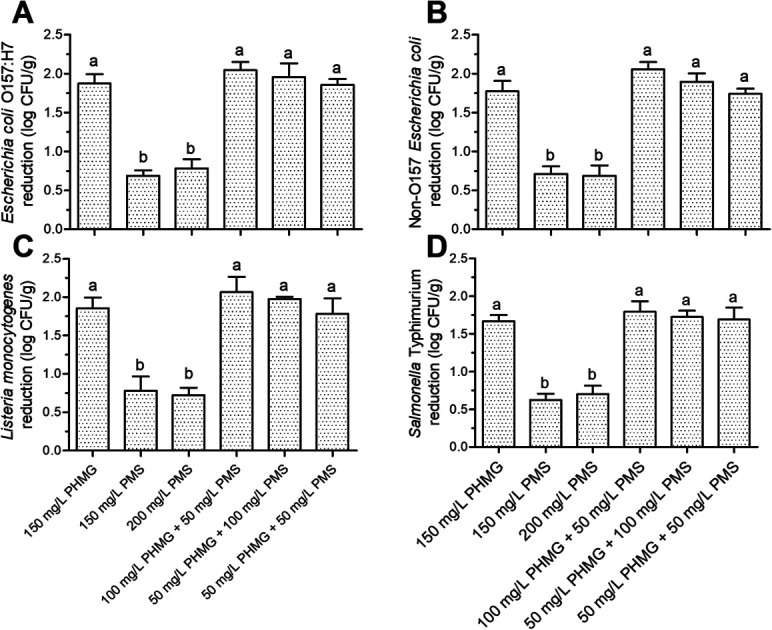

## Introduction

With gradual improvement in health awareness, more and more fresh vegetables are being consumed worldwide.^1^ Although fresh produce without thermal processing is rich in nutritional value, its consumption is bound to increase the risk of foodborne pathogen infection.^2^ For example, from 1998 to 2008, 46% of foodborne diseases in the United States were caused by consumption of contaminated fresh produce.^3^ More specifically, Callejon *et al.*^4^ reported that 32% and 43% of all foodborne diseases caused by the consumption of pathogen-contaminated vegetables in the United States were from salads and leafy vegetables, respectively; these proportions were lower, but still notable, at 27% and 22%, respectively, in Europe. The situation appears to be worse in developing countries. In Brazil, 53.1% of ready-to-eat vegetables were found to be contaminated by *Escherichia coli*, followed by *Listeria* spp. and *Salmonella* spp. at 3.7% and 1.2%, respectively.^5^ In Rwanda, 15% of vegetables were contaminated by foodborne pathogens, with *E. coli* again accounting for the largest proportion, reaching 6.1%.^6^ Therefore, it is necessary to carry out disinfection treatment before eating fresh produce to lower the risk of foodborne disease outbreaks.

Several technologies suitable for minimal processing industry applications have been developed for disinfection in recent years, including cold plasma, pulsed light, biophages, and modified atmosphere packaging.^7,8^ Despite the good results obtained using these methods, these technologies are limited for use at the ready-to-eat stage, which requires low-cost, convenient, nontoxic, and consumer-friendly methods.^9^ This is especially relevant in developing countries, which have a low rate of consuming minimally processed vegetables; thus, it is very important to offer a low-cost method to disinfect vegetables at the ready-to-eat stage.

As a novel and safe disinfectant, polyhexamethylene guanidine hydrochloride (PHMG) was approved as a food sanitizer (*i.e.*, it is deemed to be safe for use in direct contact with the food material) by the National Health Commission of the People's Republic of China.^10^ We previously reported the good disinfection efficacy of 150 mg L^−1^ PHMG against *Listeria monocytogenes*, *Escherichia coli* O157:H7, aerobic mesophilic counts (AMC), aerobic psychrotrophic counts (APC), and molds and yeasts (M&Y) on ready-to-eat lettuce after 5 min treatment, which was significantly higher than that of typical household food sanitizers (vinegar: 1% acetic acid; kettle descaler: 1% citric acid; “84” disinfectant: 200 mg L^−1^ sodium hypochlorite), and did not negatively affect the quality of the lettuce when compared with tap water treatment.^2^ However, the cost of PHMG is relatively high. Therefore, a low-cost alternative that can reach a similar or higher disinfection efficacy in combination with PHMG could promote its practical application, even in low-income countries.

Potassium peroxymonosulfate (PMS) is a white and odorless powder, which acts as a strong oxidizing compound that can degrade organic matter (*e.g.*, creatinine, chlorinated creatinine products, and arginine^11^) in water, and has also been shown to disinfect pathogens (*e.g.*, *E. coli* and *Bacillus subtilis*^11,12^) and viruses^13,14^ in water. PMS was also approved as a food sanitizer by the National Health Commission of the People's Republic of China.^10^ Importantly, the cost of PMS is approximately 1800–2200 US dollars per ton, whereas the cost of PHMG is approximately 20 000–24 000 US dollars per ton. Therefore, the objective of the present study was to investigate whether using PMS to partially replace PHMG can improve the disinfection efficiency of PHMG or obtain a similar disinfection effect. Green leaf lettuce, as a typical representative of salads and leafy vegetables, was selected as a model vegetable for this experiment.

## Materials and methods

### Sample preparation

Green leaf lettuce (*Lactuca sativa* L. var. ‘crispa’; moisture content of 96.32% ± 1.54%), without mechanical damage, was purchased from a local market on the day of the experiment. After removing the outer leaves, inner baby leaves, stem, and dirt (by rinsing under tap water for 30 s), the remaining parts were cut into pieces (diameter 5.2 × 10^−2^ m) using a circle knife.^2,15^ Each piece was then divided into four parts and drained using a sterilized (75% ethanol) manual salad spinner.

### Inoculation

Inoculation of test pathogens was performed as described previously.^2,16^ In brief, a single colony of *E. coli* O157:H7 (NCTC12900), *L. monocytogenes* (ATCC19115), non-O157 *E. coli* (ATCC25922), and *Salmonella typhimurium* (ATCC14028) was respectively inoculated in nutrition broth (Hopebio, Qingdao, China) and shaken overnight at 37 °C to prepare a working culture. The culture was adjusted to approximately 10^9^ colony-forming units (CFU) mL^−1^ and 5 mL of this culture was added to 200 mL of sterilized 0.85% sodium chloride (NaCl) solution in a sterilized plastic sampling bag. The lettuce sample (10 g) was placed in the bag and manually massaged for 20 min.^17^ The sample was then placed in a sterilized plastic tray in a biosafety cabinet for air drying, followed by washing.

### Disinfection

The treatment groups were 150 mg L^−1^ PHMG, 150 mg L^−1^ PMS, 200 mg L^−1^ PMS, 100 mg L^−1^ PHMG + 50 mg L^−1^ PMS, 50 mg L^−1^ PHMG + 100 mg L^−1^ PMS, and 50 mg L^−1^ PHMG + 50 mg L^−1^ PMS, and the control group was left untreated. PHMG and PMS were provided by Shijiashike Co. Ltd. (Liaoyang, China) and were diluted to the experimental concentration using tap water (23 ± 1 °C). A 10 g lettuce sample was immersed in the sanitizer at a ratio of 1 : 20 for 5 min. After disinfection, the sample was immersed in tap water for 30 s and drained using a sterilized salad spinner for subsequent microbial and quality analysis.

### Microbiological analysis

Each 10 g lettuce sample was transferred to a stomacher bag containing 150 mL sterilized 0.85% NaCl solution and homogenized for 90 s. A bacterial suspension series (0.1 mL) was prepared and surface-plated on modified sorbitol MacConkey agar (Hopebio), Listeria chromogenic agar (Land Bridge, Beijing, China), eosin methylene blue agar (Hopebio), and xylose lysine deoxycholate agar (Hopebio) to analyze *E. coli* O157:H7, *L. monocytogenes*, non-O157 *E. coli*, and *Salmonella typhimurium*, respectively. All plates were incubated for 24 h at 37 °C. To detect naturally present microbes, a 1 mL bacterial suspension was pour-plated onto plate count agar and incubated at 37 °C for 2 days to obtain the AMC and at 7 °C for 10 days to obtain the APC. In addition, 0.1 mL of the diluted bacterial suspension was surface-plated on Rose Bengal agar (Hopebio) and incubated at 30 °C for 3 days to quantify M&Y.

### Color analysis

Ten pieces of lettuce were randomly selected from each group, and two sites per piece were analyzed for a total of 20 readings per replicate. The color values of lightness (L*), red/green (a*), and blue/yellow (b*) were analyzed using a colorimeter (CR400; Konica Minolta, Osaka, Japan) with an aperture diameter of 11 mm. The illuminant was D65, and the color space used was a CIELab system. Before use, the colorimeter was calibrated using a white standard plate (*Y* = 82.80, *x* = 0.3194, *y* = 0.3264).

### Electrolyte leakage analysis

Electrolyte leakage was analyzed according to the method of Wang *et al.*^18^ Lettuce samples (5 g) were immersed in 250 mL distilled water for 20 s to remove the sanitizer residue and then placed in 150 mL of distilled water. After standing for 30 min, the conductivity was measured and then the samples were placed at −20 °C for 12 h. The samples were taken out to naturally thaw at room temperature, and the conductivity was measured again. Electrolyte leakage was calculated using the following formula:Electrolyte leakage (%) = Conductivity_30 min_/Conductivity_12 h_

### Sensory analysis

Crispness, color, flavour, and off-odor were evaluated on a 3-point scale,^19^ in which 0 is very bad (not characteristic of the product), 5 is the acceptability threshold, and 10 represents very good product characteristics.^18^ The samples were placed on trays with marks at the bottom, and the trays were randomly reorganized to minimize subjectivity and ensure test accuracy. Fifteen panelists aged 22–43 years from Liaoyang, Liaoning, China were invited to the sensory room, equipped with red and white lights. During the evaluation, the white light was turned on and only one person was allowed to enter the room at a time. For flavour evaluation, participants gargled for at least 30 s with distilled water (20 °C ± 1 °C) three times after tasting.

### Statistical analysis

Each experiment was performed three times. Differences between group means were evaluated by one-way analysis of variance using SPSS v.20 (IBM Inc., Armonk, NY, USA), and differences (*P* < 0.05) in mean values were analyzed by post-hoc Duncan's multiple range tests.

## Results and discussion

### Effects of adding PMS on quality properties

**Fig. 1:**
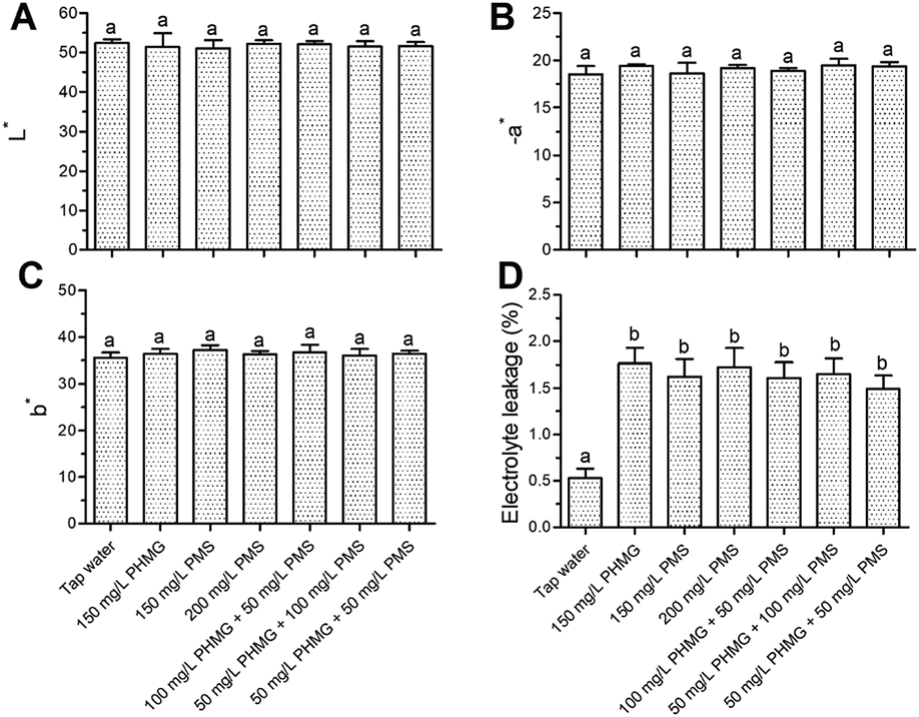
Effects of PHMG, PMS, and their combination on instrumental color (A–C) and electrolyte leakage (D) of ready-to-eat lettuce. Bars show means and standard deviations, and different letters above the columns indicate significant differences (*P* < 0.05). PHMG: polyhexamethylene guanidine hydrochloride; PMS: potassium peroxymonosulfate.

**Fig. 2:**
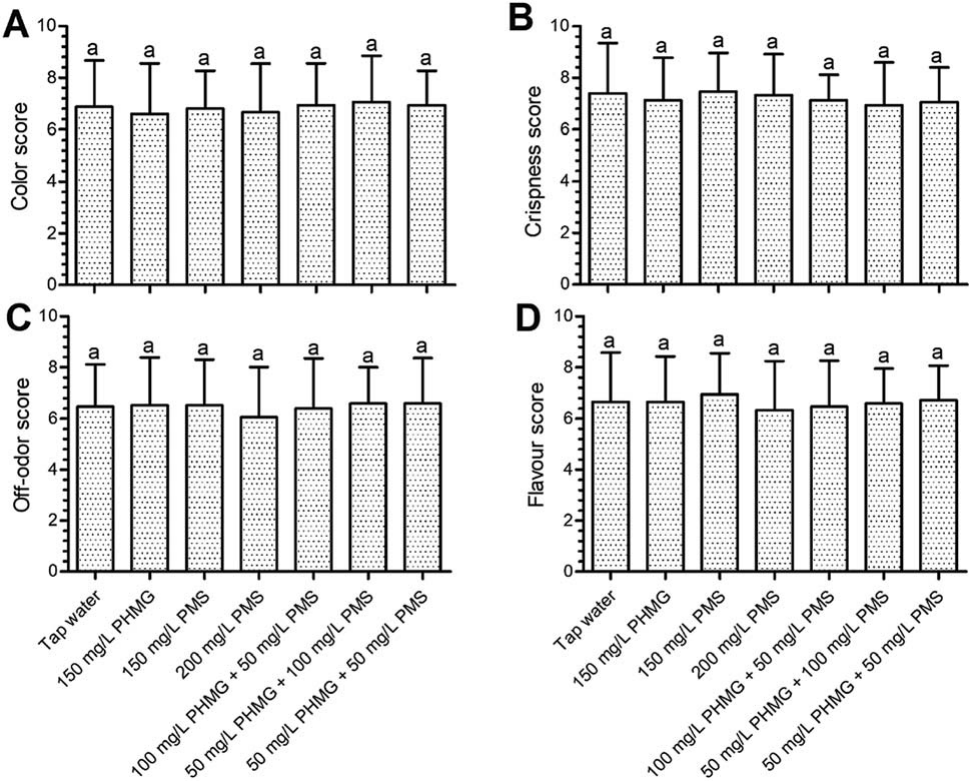
Effects of PHMG, PMS, and their combination on sensory color (A), crispness (B), off-odor (C), and flavour (D) of ready-to-eat lettuce. Bars show means and standard deviations, and different letters above the columns indicate significant differences (*P* < 0.05). PHMG: polyhexamethylene guanidine hydrochloride; PMS: potassium peroxymonosulfate.

The color index (L*, a*, and b*) of lettuce was not negatively affected by PHMG, PMS, or their combination (Fig. 1A–C). Changes in electrolyte leakage can reflect the extent of damage after disinfection.^18,20^ PHMG alone caused 1.76% electrolyte leakage (Fig. 1D), which was similar to our previous study.^2^ Electrolyte leakage was similar with the combination of PMS and PHMG, indicating that PMS did not cause additional lettuce surface damage compared with PHMG. The color was also deemed to not be negatively affected by the treatments in sensory analysis (Fig. 2A), which was consistent with the results from instrument color analysis. In addition, the off-odor, flavour, and crispness scores were also not negatively affected by the disinfectants, alone or in combination, when compared with tap water treatment (Fig. 2B–D). Collectively, these results indicated that the combined use of PHMG and PMS did not negatively affect the sensory quality of the lettuce. However, other sanitizers that are commonly used during the ready-to-eat stage, such as vinegar, have a negative impact on sensory quality. Vijayakumar and Wolfhall^21^ showed that sensory scores, including overall acceptability, off-odor, and texture, were significantly reduced in fresh-cut lettuce disinfected with white vinegar compared with those of lettuce disinfected using lemon juice and bleaching powder.

### Effects of PMS addition to the disinfection efficacy of PHMG

**Fig. 3:**
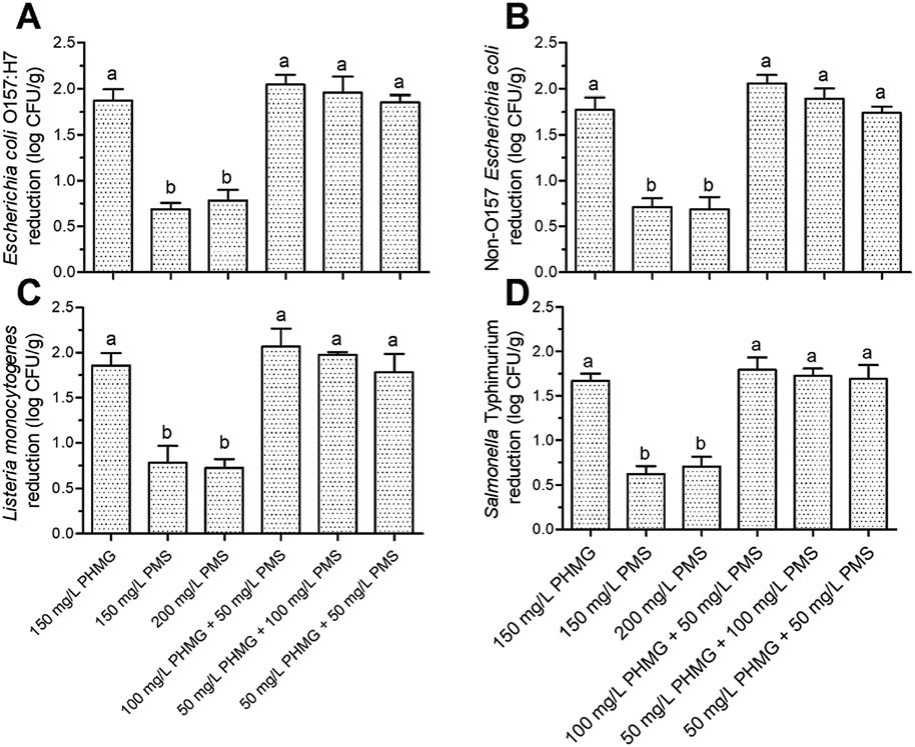
Microbial reduction (log CFU g^−1^) of *Escherichia coli* O157:H7 (A), non-O157 *Escherichia coli* (B), *Listeria monocytogenes* (C), and *Salmonella typhimurium* (D) present on ready-to-eat lettuce. The column indicates mean ± standard deviation, and the different letters above the columns indicate significant differences (*P* < 0.05). PHMG: polyhexamethylene guanidine hydrochloride; PMS: potassium peroxymonosulfate.

The counts of *E. coli* O157:H7, non-O157 *E. coli*, *L. monocytogenes*, and *Salmonella typhimurium* in the control group were 6.76 ± 0.11, 6.81 ± 0.10, 6.69 ± 0.22, and 6.72 ± 0.16 log CFU g^−1^, respectively. After disinfection with 150–200 mg L^−1^ PMS, the microbial reduction of all pathogens did not exceed 1 log CFU g^−1^ (Fig. 3A–D), which was a significantly weaker (*P* < 0.05) effect than that of PHMG, indicating that using PMS alone is not an appropriate strategy to disinfect fresh produce. However, when PHMG and PMS were combined under the same dose of PHMG alone (*i.e.*, 100 mg L^−1^ PHMG + 50 mg L^−1^ PMS or 50 mg L^−1^ PHMG + 100 mg L^−1^ PMS *vs.* 150 mg L^−1^ PHMG), the microbial reduction of *E. coli* O157:H7, non-O157 *E. coli*, *L. monocytogenes*, and *Salmonella typhimurium* was 1.96–2.05, 1.87–1.95, 1.98–2.07, and 1.72–1.79 log CFU g^−1^, respectively, which were similar to the reduction caused by PHMG alone (Fig. 3A–D). This result indicated that using PMS to partially replace PHMG can achieve the same disinfection effect as PHMG alone, and that PHMG exerts the main disinfection effect when used in combination with PMS. Interestingly, the combination of 50 mg L^−1^ PHMG + 50 mg L^−1^ PMS also showed a consistent disinfection effect on the four pathogens to that of 150 mg L^−1^ PHMG (Fig. 3A–D).

The main antibacterial mechanism of PHMG is the collapse of the outer membrane structure, leading to the formation of a pore across the membrane and subsequent DNA damage.^22,23^ By contrast, the antibacterial mechanism of action of PMS is to destroy the cell membrane, causing leakage of the cellular components and changing the cell morphology.^12^ Therefore, the combined use of PHMG and PMS may further accelerate damage to the cell membrane. However, according to our previous study, the microbial reduction of *L. monocytogenes* and *E. coli* O157:H7 caused by 150 mg L^−1^ PHMG was similar to that of 200 mg L^−1^ PHMG.^2^ Therefore, we hypothesize that with the combination of 50 mg L^−1^ PHMG and 50 mg L^−1^ PMS, the extent of damage to the cell membrane reached the limit; thus, increasing the dose (150 mg L^−1^ PHMG, 100 mg L^−1^ PHMG + 50 mg L^−1^ PMS, and 50 mg L^−1^ PHMG + 100 mg L^−1^ PMS) resulted in similar microbial reduction.

**Fig. 4:**
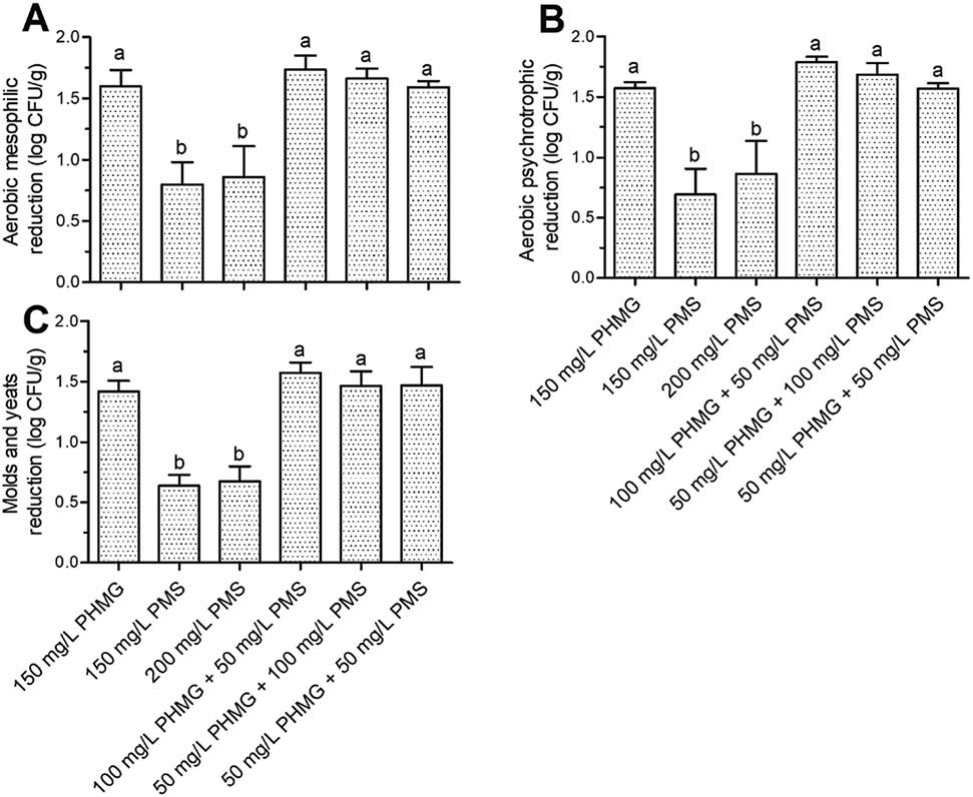
Microbial reduction (log CFU g^−1^) of aerobic mesophilic counts (A), aerobic psychrotrophic counts (B), and molds and yeasts (C) present on ready-to-eat lettuce. The column indicates mean ± standard deviation, and the different letters above the columns indicate significant differences (*P* < 0.05). PHMG: polyhexamethylene guanidine hydrochloride; PMS: potassium peroxymonosulfate.

The AMC, APC, and M&Y counts in the control group were 6.62 ± 0.35, 6.73 ± 0.36, and 5.59 ± 0.13 log CFU g^−1^, respectively. Disinfection with the combined treatment of PHMG and PMS caused equivalent reduction of these microbes to that observed with PHMG alone, although the disinfection efficacy of PMS was again significantly lower (*P* < 0.05) than that of PHMG (Fig. 4A–C).

A variety of microbial species is naturally present on fresh lettuce; thus, different disinfection methods will result in reduction of different types of aerobic mesophilic bacteria according to their cell structure characteristics. Therefore, one potential reason for the similar effects of PHMG and PMS in combination and PHMG alone is that both of these disinfectants mainly act on the cell membrane, which is present in any microbial species. Taken together, our results suggest that the combined use of PHMG and PMS can achieve consistent disinfection effects as those obtained with PHMG but at a lower cost, offering potential for application of this strategy in more low-income areas.

## Conclusions

During the ready-to-eat stage, quality properties are the main factors determining consumer acceptance of fresh produce. Our results suggest that the combined use of PHMG and PMS will not negatively affect the color (L*, a*, and b*) and sensory quality (color, off-odor, flavour, and crispness) of lettuce when compared with tap water treatment. Although the disinfection efficacy of PMS was lower than that of PHMG, when combined, the disinfection efficacy was consistent with PHMG under the same disinfection dose. PHMG clearly exerted the main disinfection effects in the combination. Moreover, even when the combined dosage of PHMG and PMS was lower than that of PHMG, similar disinfection effects were observed. In this regard, we propose the hypothesis that regardless of using a high-dose or low-dose combination, the extent of damage to the cell membrane would reach the limit, resulting in a similar degree of microbial reduction. Future research will focus on further revealing this interesting phenomenon, such as by using scanning electron microscopy and atomic force microscopy to observe the changes in membrane morphology.

## Conflicts of interest

There are no conflicts to declare.

## Supplementary Material
